# Neonatal Factors Associated with Mortality Among Preterm Infants Admitted to Neonatal Intensive Care in a Peruvian National Hospital

**DOI:** 10.3390/healthcare13192420

**Published:** 2025-09-24

**Authors:** Rosana S. Haro-Norabuena, Javier J. Gonzales-Carrillo, Miguel A. Arce-Huamani

**Affiliations:** Programa Académico de Medicina Humana, Facultad de Ciencias de la Salud, Universidad Privada Norbert Wiener, Lima 15046, Peru; rosanaharonorambuena@gmail.com (R.S.H.-N.); javier.gonzales@uwiener.edu.pe (J.J.G.-C.)

**Keywords:** premature infant, newborn intensive care units, mortality, risk factors

## Abstract

**Background/Objectives**: Preterm birth is a leading cause of neonatal mortality, especially in low- and middle-income countries. Despite advances in neonatal care, mortality among preterm infants in intensive care units remains high, and specific risk factors are not fully understood. This study aimed to identify neonatal factors associated with mortality among preterm infants admitted to the neonatal intensive care unit (NICU) of a Peruvian national hospital. **Methods**: An analytical cross-sectional study was conducted at Guillermo Almenara National Hospital in Lima, Peru, including all preterm neonates (<37 weeks gestational age) admitted to the NICU in 2022. Clinical and demographic data were extracted from medical records. Bivariate and multivariate logistic regression analyses identified independent associations with in-hospital mortality. **Results**: A total of 300 preterm neonates were included, with an in-hospital mortality rate of 15%. In adjusted analysis, extremely low birth weight (<1000 g) was the strongest predictor of mortality. Mild and severe depression in Apgar score at 1 min were associated with increased risk of death (adjusted OR: 12.08 and 6.18, respectively). Hypoglycemia was also independently associated with higher mortality (adjusted OR: 5.65). Conversely, perinatal asphyxia was linked to a lower risk of death in the multivariate model. Sex, mode of delivery, and other neonatal complications were not significant predictors after adjustment. **Conclusions**: Extremely low birth weight, abnormal Apgar scores at 1 min, and hypoglycemia are key determinants of mortality in preterm infants in intensive care. Early risk identification and focused management are essential to reducing preventable deaths in similar resource-limited settings.

## 1. Introduction

Preterm birth remains a major global health challenge: in 2020, an estimated 13.4 million babies, about 1 in 10 births, were born preterm, and nearly 1 million children died due to complications of prematurity [1,2]. Despite advances in perinatal and neonatal care, morbidity and mortality remain substantial in many low- and middle-income settings, with marked within-country disparities [3,4]. Outcomes are driven by gestational age, birth weight, and timely access to quality care, concentrating the highest mortality risk among the most vulnerable groups [5]. At the country level, Peru’s neonatal mortality remains a public health priority: in 2022, the neonatal mortality rate was ≈8 deaths per 1000 live births [6]. This work aligns with global targets under the Sustainable Development Goals, specifically SDG Target 3.2, to reduce neonatal mortality to ≤12 per 1000 live births by 2030 [7]. It is also consistent with WHO/UNICEF calls to accelerate progress on prematurity outlined in Born Too Soon: Decade of Action on Preterm Birth [2].

However, important knowledge gaps remain regarding the specific risk factors and mechanisms underlying mortality among preterm infants admitted to neonatal intensive care, particularly in resource-constrained environments [8]. While lower gestational age and birth weight are established predictors, the contribution of modifiable clinical practices and health-system characteristics (e.g., quality of care, diagnostic capacity, and resource allocation) is less clear [9]. Although global trends indicate overall reductions in preterm mortality, some regions continue to report high or rising rates despite the adoption of evidence-based interventions such as Kangaroo Mother Care and breastfeeding support [1,3]. Persistent gaps are partly explained by differences in infrastructure, workforce, access to diagnostics and treatment, and variations in perinatal management. These system-level constraints limit the consistent translation of proven interventions into improved outcomes [4,8]. Sociodemographic disadvantages, including low maternal education and rural residence, further increase risk. Inadequate antenatal care is an additional independent contributor [10,11].

These gaps underscore the need to identify, in a comprehensive and context-specific manner, the factors associated with neonatal mortality among preterm infants, particularly in low-resource and Latin American settings, to inform effective, locally appropriate strategies to reduce preventable deaths.

Beyond neonatal factors, specific pregnancy pathologies contribute to early delivery and adverse neonatal outcomes. Placenta accreta spectrum (PAS) often necessitates iatrogenic preterm birth because of the risk of peripartum hemorrhage and the need for scheduled delivery in specialized centers; when emergency delivery is required, neonatal morbidity is higher. Recent data demonstrate worse neonatal outcomes after emergent cesarean compared with planned management in PAS, reinforcing the link between PAS and prematurity-driven risk [12].

Therefore, the objective of the present study was to identify the neonatal factors associated with mortality among preterm infants admitted to the neonatal intensive care unit in a Peruvian national hospital.

## 2. Materials and Methods

### 2.1. Study Design and Setting

This analytical cross-sectional study was conducted in the neonatal intensive care unit (NICU) of Guillermo Almenara National Hospital, a tertiary-level referral center in Lima, Peru. The objective was to identify neonatal factors associated with mortality among preterm infants. The study covered a 12-month period (1 January–31 December 2022). The hospital serves a diverse urban and peri-urban population and is a national reference center for high-risk neonatal care. The study period refers to the window of medical records reviewed; data abstraction and analysis began only after institutional ethics approval in 2024 (Approval No. 0045-2024).

### 2.2. Population and Sample

**Figure 1:**
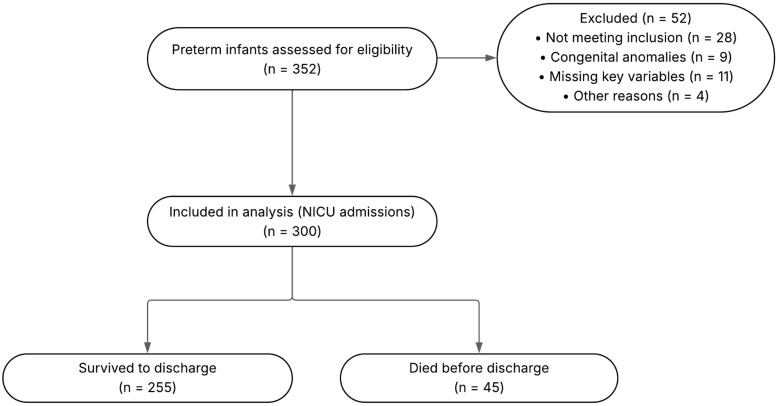
Study flow diagram of participant selection.

The target population consisted of all preterm neonates, defined as live-born infants with a gestational age of less than 37 weeks, who were admitted to the NICU of Guillermo Almenara National Hospital during the study period. Inclusion criteria required neonates to have complete clinical records, be born at the hospital, and be hospitalized in the NICU within the first hours of life. Exclusion criteria were established to minimize confounding and included neonates referred to from other healthcare facilities, those with a diagnosis of stillbirth, preterm infants not admitted to the NICU, and neonates born to mothers with laboratory-confirmed COVID-19 infection. The study flow, including the numbers assessed, excluded with reasons, and included, as well as discharge outcomes, is summarized in Figure 1.

Sample size determination followed rigorous methodological standards for analytical observational studies, employing a two-sided significance level of 95% and a statistical power of 80%. Parameters were based on data from a previous study of neonatal outcomes in similar populations [13]. The calculation assumed equal allocation between exposed and non-exposed groups, with expected outcome frequencies of 31% and 16%, respectively, and an anticipated odds ratio of 2.3. The design also incorporated a risk ratio of 1.9 and an absolute risk difference of 14%. Consequently, the final sample comprised 300 neonates, ensuring sufficient statistical power to detect clinically meaningful associations in neonatal outcomes.

### 2.3. Data Collection

Data were abstracted from medical records using a structured chart-abstraction form developed specifically for this study. Content validity was established through expert review by four neonatology/pediatrics specialists who rated clarity, pertinence, and relevance (Kendall’s W = 0.918; *p* = 0.002) [14]. A pilot test demonstrated good reliability (Cronbach’s α = 0.892) [15]. The form captured demographic and clinical characteristics as well as neonatal outcomes for all preterm infants meeting the inclusion criteria.

### 2.4. Variables

The primary outcome of this study was in-hospital neonatal mortality, defined as the death of a preterm infant within the first 28 days of life during admission to the neonatal intensive care unit. This definition aligns with established international guidelines for neonatal epidemiology and allows direct comparison with published literature. Independent variables were selected based on their clinical relevance and previous evidence of association with neonatal outcomes. These included infant sex, mode of delivery (classified as vaginal or cesarean section), and detailed categories of birth weight [16]: extremely low birth weight (less than 1000 g), very low birth weight (1000 to 1499 g), low birth weight (1500 to 2499 g), and adequate weight for gestational age. Gestational age was verified according to last menstrual period and/or early obstetric ultrasound, consistent with local and international standards.

Apgar score at 1 min [17] was recorded and reported using standard categories: 7–10 (reassuring/normal), 4–6 (moderately abnormal), and 0–3 (low/severe). Additional clinical variables were systematically collected and included the presence or absence of neonatal infection [18] (encompassing both early- and late-onset sepsis), perinatal asphyxia [19] (diagnosed on the basis of clinical and laboratory criteria), hypothermia at birth [20], intraventricular hemorrhage (confirmed by cranial imaging), neonatal jaundice [21] (requiring medical intervention), hypoglycemia [22] (based on age-appropriate plasma glucose thresholds), respiratory distress syndrome [23], transient tachypnea of the newborn [24], and bronchopulmonary dysplasia [25]. All variables were operationalized according to widely recognized clinical definitions, ensuring both local validity and comparability with global research.

Gestational age was evaluated for inclusion in multivariable analyses; however, it was not retained in the final model due to strong collinearity with birth weight and model instability when both were entered simultaneously. Birth weight was prioritized as the clinically interpretable indicator of fetal maturity and risk, avoiding redundancy and unstable estimates.

The registry captured in-hospital status at discharge (alive or dead); age at death was not recorded, so the timing of deaths (early: 0–6 days; late: 7–27 days) could not be derived.

### 2.5. Data Analysis

All collected data were entered into a dedicated research database and subsequently exported to Stata version 18.0 (StataCorp LLC, College Station, TX, USA) for statistical analysis. The statistical approach was chosen to maximize the validity and interpretability of the results, reflecting current international standards for observational neonatal research.

Descriptive statistics were calculated for the entire cohort. Categorical variables were reported as absolute frequencies and percentages, while continuous variables were summarized as medians and interquartile ranges due to non-normal data distributions, which were assessed with the Kolmogorov–Smirnov test [26]. The initial bivariate analysis was performed to explore associations between each independent variable and the outcome of neonatal mortality. For categorical variables, associations were evaluated using the chi-square test or Fisher’s exact test when expected frequencies were low; for continuous variables, the Mann–Whitney U test was employed.

Importantly, variables considered for inclusion in the multivariate model were selected based on their statistical association with mortality in the bivariate analysis, using a liberal threshold of *p* ≤ 0.20, as well as their recognized importance in international scientific literature. This dual criterion is widely recommended in epidemiological modeling to balance statistical rigor with clinical plausibility and to minimize residual confounding. However, variables with at least one category showing complete separation where no events of mortality occurred were excluded from the final model. Specifically, both “neonatal infection” and “hypothermia” had no deaths in the “No” category, leading to zero cell frequencies that precluded reliable estimation of odds ratios in logistic regression. The inclusion of such variables can yield non-estimable coefficients, infinite odds ratios, and model instability, thereby affecting both the statistical and clinical interpretation of the results.

Accordingly, the final multivariate logistic regression model included only those variables that demonstrated sufficient outcome variability and clinical relevance. These comprised infant sex, birth weight (with categories regrouped to avoid those with no events), Apgar score at 1 min, perinatal asphyxia, and hypoglycemia. Intraventricular hemorrhage was also included in the modeling process based on its established clinical significance, despite a marginal *p*-value in bivariate analysis. The modeling approach ensures that all reported odds ratios are both statistically interpretable and clinically meaningful, in keeping with accepted international recommendations for observational epidemiology.

Missing data were handled by complete-case analysis. Records with missing key variables were excluded, as shown in the study flow diagram, and no statistical imputation was performed. Denominators in tables reflect available data for each variable.

Adjusted and crude odds ratios with 95% confidence intervals were estimated for each predictor, and a two-sided *p*-value < 0.05 was considered statistically significant. The presence of wide confidence intervals for some categories reflects the relatively small number of events in these subgroups and underscores the need for cautious interpretation of these specific estimates. Nevertheless, the direction and magnitude of the observed associations were highly consistent with previous studies and with biological plausibility, which strengthens the external validity and relevance of the findings.

### 2.6. Ethical Considerations

The protocol was reviewed and approved by the Ethics Committee of Universidad Privada Norbert Wiener (Approval No. 0045-2024) and received formal authorization from the Department of Neonatology at Guillermo Almenara National Hospital. Because this was a retrospective review of medical records, there was no direct patient or family contact. Confidentiality and anonymity were safeguarded by assigning unique alphanumeric codes and restricting database access to authorized personnel. All procedures complied with the Declaration of Helsinki and applicable national and institutional regulations governing the use of health data for research. This approval explicitly covered records from January–December 2022; no data were collected, abstracted, or analyzed prior to approval.

### 2.7. Data Availability and Use of Generative AI

All data, materials, and protocols are available upon reasonable request to the corresponding author. No generative artificial intelligence (GenAI) tools were used in study design, data collection, analysis, or interpretation.

## 3. Results

### 3.1. Descriptive Analysis of Results

A total of 300 preterm neonates were included (Figure 1). Baseline characteristics are summarized in Table 1. Overall, the cohort reflects a high-risk NICU population, with a predominance of cesarean delivery, a majority within low-birth-weight strata, and frequent early complications. The in-hospital mortality rate was 15% (Table 1). Timing of deaths (early vs. late) could not be reported because age at death was not available in the registry.

### 3.2. Bivariate Analysis

In bivariate analyses, mortality was associated with extremely low birth weight, 1-min Apgar depression, infection, perinatal asphyxia, hypothermia, and hypoglycemia. No associations were observed for sex, mode of delivery, intraventricular hemorrhage, neonatal jaundice, respiratory distress syndrome, transient tachypnea of the newborn, or bronchopulmonary dysplasia (Table 2).

### 3.3. Multivariate Analysis

In the multivariable model, very low and low birth weight had lower odds of death relative to extremely low birth weight; mild and severe 1-min Apgar depression and hypoglycemia were independently associated with higher mortality; perinatal asphyxia showed a lower-adjusted odds; sex was not significant (Table 3).

**Figure 2:**
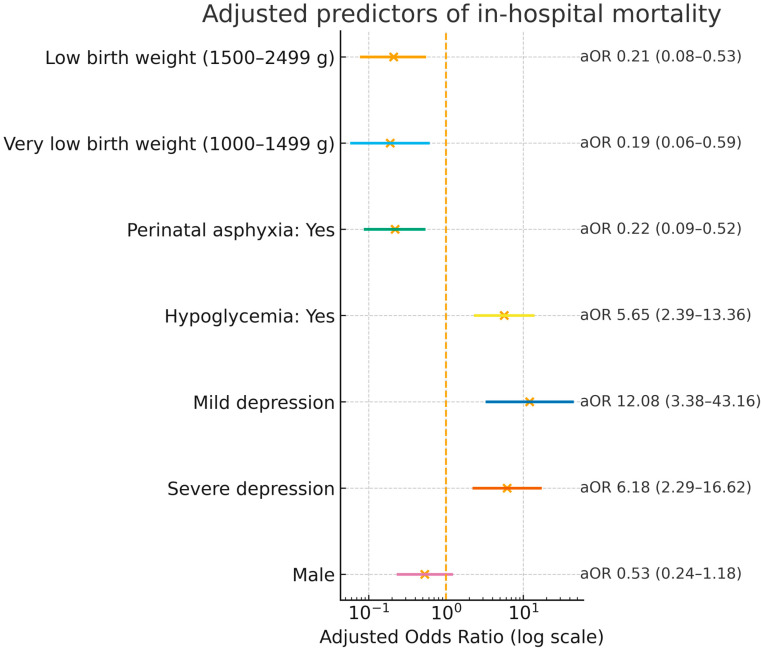
Adjusted odds ratios (aOR) for in-hospital mortality (log scale).

Points represent aORs and horizontal bars the 95% confidence intervals. Reference categories: extremely low birth weight (<1000 g), normal 1-min Apgar, no perinatal asphyxia, no hypoglycemia, and female sex (Figure 2).

## 4. Discussion

Mortality among preterm neonates in NICU care is closely tied to biological vulnerability and acute clinical instability. The high prevalence of low and extremely low birth weight reflects the ongoing burden of prematurity in resource-limited settings, where advanced neonatal support is constrained. Abnormal 1-min Apgar scores are strongly associated with death, underscoring the need for rapid resuscitation and close monitoring, and hypoglycemia likewise correlates with higher mortality, highlighting the value of early detection and treatment. Although perinatal asphyxia was frequent, its inverse association with mortality in adjusted analyses likely reflects confounding and co-occurring conditions rather than a protective effect. The in-hospital mortality rate of 15% indicates substantial room for improvement and supports prioritizing evidence-based strategies for risk stratification, stabilization, and continuous care for preterm infants.

Extremely low birth weight (ELBW) (<1000 g) was the strongest correlate of in-hospital mortality, whereas very low and low birth weight showed substantially lower adjusted risks. This pattern aligns with the literature, although few studies report ELBW-specific adjusted estimates. For example, Bogale et al. [27] (Ethiopia) did not report an adjusted effect for ELBW and noted that deaths clustered in the 1000–1499 g range; Mekasha et al. [28] (Ethiopia) found higher mortality with low birth weight; Tembo et al. [29] (Zambia) observed excess mortality across lower-birth-weight strata; Aguma et al. [30] (Uganda) reported markedly elevated risk among very low-birth-weight infants. Limited ELBW-specific reporting likely reflects cohort composition and survival differences across settings. Collectively, these findings underscore the central role of birth weight, especially ELBW, in preterm mortality and the need for targeted interventions in resource-limited units.

Both mild and severe depression in the 1-min Apgar score were independently and strongly associated with higher in-hospital mortality among preterm neonates. Direct comparisons at 1 min are limited because most studies emphasize the 5-min score: Bogale et al. [27] (Ethiopia), Mekasha et al. [28] (Ethiopia), and Tembo et al. [29] (Zambia) did not report the 1-min Apgar as a significant adjusted predictor, whereas Aguma et al. [30] (Uganda) showed that a 5-min Apgar < 7 conferred a 4.5-fold higher odds of death (aOR 4.52, 95% CI 2.04–10.01), and Mikael Norman et al. [31] found higher 5- and 10-min scores to be protective. Taken together, the literature’s directionality aligns with our findings, reinforcing the clinical importance of early postnatal assessment and prompt identification of infants with early depression, even if most prior reports focus on the 5-min score.

Hypoglycemia independently predicted in-hospital mortality among preterm neonates in our cohort. This aligns with Alemu Bogale et al. [27], who reported a significant association (AOR = 2.85, 95% CI 1.09–7.44). In contrast, Deborah Tembo et al. [29] found that a primary diagnosis of glycemic disturbance (hyperglycemia or hypoglycemia) was associated with reduced mortality rate ratios, and Lire Lemma Tirore et al. [32] noted high hypoglycemia prevalence but no independent effect in their final multivariable model. Differences likely reflect heterogeneity in definitions, measurement timing, and management protocols across settings. Nevertheless, our findings, together with Bogale et al., support early detection and aggressive treatment of hypoglycemia, particularly in resource-limited units where preventable deaths remain high.

Perinatal asphyxia showed an inverse association with in-hospital mortality after adjustment in our cohort, despite its high frequency. This contrasts with reports showing higher risk: Abebaw et al. [33] (AHR 1.74, 95% CI 1.01–2.76), Mohamed et al. [34] (AOR 2.40, 95% CI 1.26–4.43), and Toma et al. [35] (AHR 2.44, 95% CI 1.33–4.49). By contrast, Bogale et al. [27] did not find an independent association after adjustment. Differences likely reflect variation in case definitions and severity grading, resuscitation and subsequent management, and collinearity with co-occurring perinatal risks, underscoring the need for standardized criteria and careful adjustment in future studies.

The inverse association between perinatal asphyxia and mortality in our adjusted model likely reflects methodological rather than biological effects. First, misclassification is possible because our operational definition combines clinical and laboratory criteria that may vary in documentation and severity across cases. Second, collinearity with closely related variables, most notably 1-min Apgar and birth weight, can produce suppression effects that attenuate or invert the adjusted association when entered simultaneously. Third, survival bias cannot be excluded: the most severely asphyxiated newborns may have died before NICU admission and therefore were not captured in our cohort. Taken together, these issues argue against a protective effect of asphyxia per se and support cautious interpretation of the adjusted estimate.

After adjustment, sex, mode of delivery, intraventricular hemorrhage, neonatal jaundice, hyaline membrane disease, transient tachypnea of the newborn, and bronchopulmonary dysplasia were not associated with in-hospital mortality. This pattern is consistent with prior reports: sex was not an independent predictor in cohorts by Nicholas Aguma et al. [30] and Mikael Norman et al. [31] (the latter noting a small, non-significant excess among males), and mode of delivery showed no adjusted association in analyses by Deborah Tembo et al. [29] and Ermias Abebaw et al. [33]. An exception is Amha Mekasha et al. [28], who reported higher risk among females. Overall, once major risk factors are accounted for, these variables appear to have limited prognostic value, suggesting that resources should emphasize early identification and management of more impactful determinants.

The observed in-hospital mortality rate of 15% among preterm neonates in this study falls within the broad range reported in recent studies from diverse contexts. Alemu Bogale et al. [27] described a lower preterm neonatal mortality rate of 10.9%, while Mikael Norman et al. [31] found a rate of 7.6% among a large multicenter cohort, suggesting better outcomes in certain high-resource or well-supported settings. In contrast, substantially higher mortality rates have been documented in other cohorts, such as 29.31% in the study by Amha Mekasha et al. [28], 40.2% reported by Deborah Tembo et al. (2024) [29], and 27.11% in the analysis by Ermias Abebaw et al. [33]. Additional reports by Hamda Ahmed Mohamed et al. [34], Lire Lemma Tirore et al. [32], and Temesgen Mohammed Toma et al. [35] indicate mortality rates ranging from 18.6% to 26.08%, reflecting significant variability even within similar geographic regions. Nicholas Aguma et al. [30] found a comparable rate of 17.49%, further supporting the notion that mortality remains unacceptably high for preterm neonates in many settings. This variation in mortality rates may be influenced by differences in neonatal care quality, resource availability, patient profiles, and referral patterns. The present findings highlight both the ongoing challenge of reducing neonatal deaths and the potential for improvement with targeted interventions and system-level changes, which are particularly relevant for health policy and global neonatal survival efforts.

These findings have direct clinical implications. First, extremely low birth weight infants should be prioritized for continuous cardio-respiratory and glucose monitoring, strict thermoregulation, and early escalation of support [1,2]. Second, routine hypoglycemia screening should be standardized at admission and at regular intervals during the first hours of life, with rapid treatment protocols (early feeding and, when indicated, intravenous dextrose) to prevent recurrent low glucose [27]. Third, newborns with depressed 1-min Apgar scores warrant immediate resuscitation and close early surveillance, as early physiological instability strongly predicts mortality [17].

The results of this research provide critical evidence regarding the main neonatal factors associated with mortality among preterm infants admitted to intensive care in a large Peruvian national hospital. These findings not only confirm well-established risk factors, such as extremely low birth weight and abnormal Apgar scores, but also highlight the independent impact of hypoglycemia and the complex interplay of perinatal asphyxia in this population. By employing rigorous methodological standards and context-specific definitions, this study offers valuable insights that are directly applicable to similar settings in Latin America and other low- and middle-income regions facing comparable challenges. The identification of highly prevalent and modifiable risk factors underscores the urgent need to prioritize early risk stratification, timely clinical interventions, and system-level improvements in neonatal care. Ultimately, these results can inform health policy decisions, guide the allocation of limited resources, and support the development of targeted interventions to reduce preventable neonatal deaths, contributing meaningfully to the advancement of global health equity and the achievement of Sustainable Development Goal 3 on child survival.

This study provides context-specific evidence from a tertiary national referral NICU in Peru and identifies actionable targets to reduce preventable deaths in similar units. Using a well-defined cohort of high-risk preterm infants, standardized clinical definitions, and a robust analytic approach enhanced internal and external validity. Comprehensive data collection with a validated abstraction tool minimized information bias. Limitations include the retrospective design susceptible to missing data and unmeasured confounding. Although rigorous inclusion criteria, systematic chart review, and sensitivity analyses helped mitigate these concerns, the single-center setting may limit generalizability, though the hospital’s role as a national referral center supports applicability to similar institutions. Additionally, variables with complete separation (e.g., infection and hypothermia) showed zero events in one category and were excluded from the final model to preserve estimability, which may have limited our ability to quantify their independent effects. Because age at death was not collected, we were unable to stratify mortality into early (0–6 days) and late (7–27 days) periods. As a single-center study in a national referral NICU, case mix and service organization may differ from other Peruvian or Latin American hospitals; therefore, the effect sizes and absolute risks may not generalize beyond similar tertiary units.

## 5. Conclusions

Preterm mortality in this NICU cohort was driven primarily by extremely low birth weight, depressed 1-min Apgar, and hypoglycemia. Clinically, these findings support prioritizing ELBW infants for continuous monitoring and early escalation of support, standardizing admission and serial hypoglycemia screening with rapid treatment, and ensuring immediate resuscitation and close surveillance for infants with low 1-min Apgar. For policymakers, strengthening NICU capacity (staffing, glucose monitoring and therapies, and thermoregulation) and embedding simple risk-stratification protocols can reduce preventable deaths in similar resource-limited settings. The unexpected inverse-adjusted association with perinatal asphyxia should be interpreted cautiously and investigated further, rather than used to change clinical practice. These conclusions reflect care delivered in a tertiary-level national referral NICU in Lima, Peru, and are most applicable to similar resource-constrained tertiary units.

## Figures and Tables

**Table 1:** Clinical characteristics of preterm neonates in the neonatal intensive care unit.

Characteristic	N (%)
Sex	
Female	147 (49.0)
Male	153 (51.0)
Mode of delivery	
Vaginal	42 (14.0)
Cesarean section	258 (86.0)
Birth weight (categorical)	
Extremely low birth weight (<1000 g)	54 (18.0)
Very low birth weight (1000–1499 g)	78 (26.0)
Low birth weight (1500–2499 g)	167 (55.67)
Adequate birth weight for gestational age	1 (0.33)
Birth weight (numeric) ^1^	1620.5 (IQR 1246–1923)
Apgar score at 1 min	
7–10 (reassuring/normal)	245 (81.7)
4–6 (moderately abnormal)	20 (6.6)
0–3 (low/severe)	35 (11.7)
Neonatal infection	
No	47 (15.7)
Yes	253 (84.3)
Perinatal asphyxia	
No	55 (18.3)
Yes	245 (81.7)
Hypothermia	
No	47 (15.7)
Yes	253 (84.3)
Intraventricular hemorrhage	
No	97 (32.3)
Yes	203 (67.7)
Neonatal jaundice	
No	218 (72.7)
Yes	82 (27.3)
Hypoglycemia	
No	245 (81.7)
Yes	55 (18.3)
Hyaline membrane disease	
No	79 (26.3)
Yes	221 (73.7)
Transient tachypnea of the newborn	
No	221 (73.7)
Yes	79 (26.3)
Bronchopulmonary dysplasia	
No	79 (26.3)
Yes	221 (73.7)
Mortality	
No	255 (85.0)
Yes	45 (15.0)

^1^ Median, interquartile range (IQR).

**Table 2:** Bivariate analysis of neonatal factors associated with mortality among preterm infants admitted to the neonatal intensive care unit.

Characteristic	Mortality		*p*-Value
	No	Yes	
	(n = 255)	(n = 45)	
Sex			0.201
Female	121 (47.45)	26 (57.78)	
Male	134 (52.55)	19 (42.22)	
Mode of delivery			0.744
Vaginal	35 (13.73)	7 (15.56)	
Cesarean section	220 (86.27)	38 (84.44)	
Birth weight (categorical)			<0.001 *
Extremely low birth weight (<1000 g)	33 (12.94)	21 (46.67)	
Very low birth weight (1000–1499 g)	71 (27.84)	7 (15.56)	
Low birth weight (1500–2499 g)	150 (58.82)	17 (37.78)	
Adequate birth weight for gestational age	1 (0.39)	0 (00)	
Birth weight (numeric) ^1^	1622 (IQR 1204–1920)	1590 (IQR 1344–1928)	0.843
Apgar score at 1 min			<0.001 *
Normal	222 (87.06)	23 (51.11)	
Mild depression	10 (3.92)	10 (22.22)	
Severe depression	23 (9.02)	12 (26.67)	
Neonatal infection			0.002
No	47 (18.43)	0 (0.0)	
Yes	208 (81.57)	45 (100.0)	
Perinatal asphyxia			<0.001
No	33 (12.94)	22 (48.89)	
Yes	222 (87.06)	23 (51.11)	
Hypothermia			0.002
No	47 (18.43)	0 (0.0)	
Yes	208 (81.57)	45 (100.0)	
Intraventricular hemorrhage			0.220
No	86 (33.73)	11 (24.44)	
Yes	169 (66.27)	34 (75.56)	
Neonatal jaundice			0.637
No	184 (72.16)	34 (75.56)	
Yes	71 (27.84)	11 (24.44)	
Hypoglycemia			<0.001
No	222 (87.06)	23 (51.11)	
Yes	33 (12.94)	22 (48.89)	
Hyaline membrane disease			0.497
No	69 (27.06)	10 (22.22)	
Yes	186 (72.94)	35 (77.78)	
Transient tachypnea of the newborn			0.497
No	186 (72.94)	35 (77.78)	
Yes	69 (27.06)	10 (22.22)	
Bronchopulmonary dysplasia			0.497
No	69 (27.06)	10 (22.22)	
Yes	186 (72.94)	35 (77.78)	

^1^ Median, interquartile range (IQR). *p*-value calculated using the chi-square test; for variables marked with an asterisk (*), the Fisher’s exact test was used. For continuous variables, the Mann–Whitney U test was applied.

**Table 3:** Multivariate analysis of neonatal factors associated with mortality among preterm infants admitted to the neonatal intensive care unit.

Variable	Crude OR (95% CI)	*p*-Value	Adjusted OR (95% CI)	*p*-Value
Sex				
Female	Reference		Reference	
Male	0.66 (0.35–1.26)	0.212	0.53 (0.24–1.18)	0.118
Birth weight (categorical)				
Extremely low birth weight (<1000 g)	Reference		Reference	
Very low birth weight (1000–1499 g)	0.15 (0.06–0.40)	<0.001	0.19 (0.06–0.59)	0.004
Low birth weight (1500–2499 g)	0.17 (0.08–0.37)	<0.001	0.21 (0.08–0.53)	0.001
Apgar score at 1 min				
Normal	Reference		Reference	
Mild depression	9.60 (3.62–25.50)	<0.001	12.08 (3.38–43.16)	<0.001
Severe depression	5.01 (2.21–11.38)	<0.001	6.18 (2.29–16.62)	<0.001
Perinatal asphyxia				
No	Reference		Reference	
Yes	0.16 (0.08–0.31)	<0.001	0.22 (0.09–0.52)	0.001
Hypoglycemia				
No	Reference		Reference	
Yes	6.41 (3.21–12.77)	<0.001	5.65 (2.39–13.36)	<0.001

OR: odds ratio; 95% CI: 95% confidence interval. Adjusted odds ratios are from the multivariate logistic regression model, including all variables listed.

## Data Availability

The data supporting the findings of this study are available from the corresponding author upon reasonable request.
